# Factors associated with sexual and reproductive health stigma among adolescent girls in Ghana

**DOI:** 10.1371/journal.pone.0195163

**Published:** 2018-04-02

**Authors:** Kelli Stidham Hall, Emmanuel Morhe, Abubakar Manu, Lisa H. Harris, Elizabeth Ela, Dana Loll, Giselle Kolenic, Jessica L. Dozier, Sneha Challa, Melissa K. Zochowski, Andrew Boakye, Richard Adanu, Vanessa K. Dalton

**Affiliations:** 1 Rollins School of Public Health, Emory University, Atlanta, GA, United States of America; 2 University of Allied Health and Sciences, Ho, Ghana; 3 School of Public Health, University of Ghana, Accra, Ghana; 4 Department of Obstetrics and Gynecology, University of Michigan, Ann Arbor, MI, United States of America; 5 Komfo Anokye Teaching Hospital, Kwame Nkrumah University of Science and Technology, Kumasi, Ghana; University of Westminster, UNITED KINGDOM

## Abstract

**Objective:**

Using our previously developed and tested Adolescent Sexual and Reproductive Health (SRH) Stigma Scale, we investigated factors associated with perceived SRH stigma among adolescent girls in Ghana.

**Methods:**

We drew upon data from our survey study of 1,063 females 15-24yrs recruited from community- and clinic-based sites in two Ghanaian cities. Our Adolescent SRH Stigma Scale comprised 20 items and 3 sub-scales (Internalized, Enacted, Lay Attitudes) to measure stigma occurring with sexual activity, contraceptive use, pregnancy, abortion and family planning service use. We assessed relationships between a comprehensive set of demographic, health and social factors and SRH Stigma with multi-level multivariable linear regression models.

**Results:**

In unadjusted bivariate analyses, compared to their counterparts, SRH stigma scores were higher among girls who were younger, Accra residents, Muslim, still in/dropped out of secondary school, unemployed, reporting excellent/very good health, not in a relationship, not sexually experienced, never received family planning services, never used contraception, but had been pregnant (all p-values <0.05). In multivariable models, higher SRH stigma scores were associated with history of pregnancy (β = 1.53, CI = 0.51,2.56) and excellent/very good self-rated health (β = 0.89, CI = 0.20,1.58), while lower stigma scores were associated with older age (β = -0.17, 95%CI = -0.24,-0.09), higher educational attainment (β = -1.22, CI = -1.82,-0.63), and sexual intercourse experience (β = -1.32, CI = -2.10,-0.55).

**Conclusions:**

Findings provide insight into factors contributing to SRH stigma among this young Ghanaian female sample. Further research disentangling the complex interrelationships between SRH stigma, health, and social context is needed to guide multi-level interventions to address SRH stigma and its causes and consequences for adolescents worldwide.

## Introduction

Stigma “deeply discredits and transforms people from whole individuals to tainted, discounted ones” [1,2]. It is a complex, contextual, dynamic social process that “marks” an individual for an attribute that violates social expectations and is devalued culturally [1,2]. Stigma scholars have described it as driver of health inequalities and a fundamental social determinant of health [3–5]. The majority of research considering the health-related causes and consequences of stigma have focused on stigma specifically occurring with mental illness, minority sexual orientation, obesity, HIV/AIDS, disability, and minority race/ethnicity [1–11].

Stigma occurring in the context of adolescent sexual behavior, pregnancy, early childbearing, abortion, and sexually transmitted infections (STIs) may lead to adverse health and social consequences, including shame, social marginalization, violence and mental health morbidity [12–21]. Our recent work has formally conceptualized and measured social stigma spanning a broader continuum of SRH events to explore whether adolescent SRH stigma at the environmental or community level negatively influences family planning decision-making and behaviors [22–24]. Based upon formative qualitative research [22,23], we developed an Adolescent SRH Stigma Scale to assess environmental stigma within the community accompanying different dimensions of SRH and family planning [24]. The 20-item instrument, which comprises Internalized Stigma, Enacted Stigma, and Stigmatizing Lay Attitudes sub-scales, was then tested in a survey study of 1,080 women ages 15–24 recruited from schools, health facilities, and universities in two cities in Ghana.

While this work was useful in providing initial estimates of SRH stigma and documenting relationships between girls’ higher SRH stigma scores and nonuse of modern contraception [22–24], findings alone are not sufficient to inform targeted, multi-level interventions to reduce or manage SRH stigma. Indeed, the causes of adolescent SRH stigma have been given little attention in research to date, but a better understanding of the factors contributing to environmental SRH stigma is needed to identify young women and communities most at risk for stigma and its adverse consequences. Such research is also needed to guide more holistic public health practices, programs and policies that ultimately de-stigmatize SRH for adolescents in Africa and elsewhere.

We investigated a diverse set of demographic, social and health factors associated with environmental SRH stigma perceived by adolescent girls from two urban communities in western Sub-Saharan Africa.

## Materials and methods

### Study design and sample

The study was approved by the Institutional Ethics Review Boards of the Ghana Health Services, University of Ghana, Kwame Nkrumah University of Science and Technology, and University of Michigan. For this analysis, we drew upon data from a comprehensive survey study of adolescent and young adult females ages 15–24 from community- and clinic-based sites in Accra and Kumasi, Ghana. Our study design and sample have been described elsewhere [24]. In brief, a cluster sampling technique was used to recruit 1,080 participants from 11 total sites: four Senior High Schools within the Ghana Educational Service, five Ghana Health Service facilities (e.g. antenatal, postnatal, family planning and child welfare clinics), and two universities. This sampling frame provided heterogeneity in types of clinics (antenatal, postnatal, family planning, adolescent, abortion, child welfare) and schools (public, co-education, female only) and the characteristics of the populations they serve (i.e. reproductive, relationship, socioeconomic, religious characteristics).

Trained Ghanaian research assistants (RAs) approached women at recruitment sites, provided study information, invitations to participate, and screened young women for eligibility. Eligible potential participants were: 1) females, 2) between the ages of 15 and 25, 3) residents of Accra or Kumasi or surrounding areas, and 4) able to speak English or one of two local languages (Ga or Twi). Following eligibility screening and enrollment, RAs obtained informed written consent; we obtained parental consent waivers from all Ghanaian IRBs given the sensitive nature of our survey and to ensure confidentiality. Enrolled participants then completed the confidential survey interview administered by RAs via tablets using Qualtrics Mobile, a secured, web-based data collection and management system. Our survey was informed by our preceding qualitative study, our prior reproductive health services and stigma-related research, an extensive review of the relevant literature on stigma and the social context of adolescent SRH, and drawing upon well-established existing survey items (e.g. Demographic and Health Survey).[1,2,6–11,22–24] Specifically, the included sections to measure girl’s: 1) demographic and social background characteristics; 2) general and mental health and social wellbeing; 3) family planning knowledge, attitudes, and perceived norms and barriers; 4) reproductive histories; 5) contraceptive and family planning service experiences; and 6) different dimensions of adolescents’ perceptions of and experiences related to SRH stigma within their environments. We pilot-tested the survey in interviews with a convenience sample of 25 adolescent and young adult women from our targeted recruitment sites to ensure comprehension. Survey completion times ranged from 30–90 minutes, which given the cumulative nature of the content was determined primarily by the extent of participants’ reproductive histories. Participants were offered a pre-paid telephone card for participating in the study, sharing their experiences and perspectives, and as appreciation for their time.

### Measures

#### Sexual and reproductive health stigma

The comprehensive survey included our new Adolescent SRH Stigma Scale, which we psychometrically tested and validated specifically for this project.[24] The final version of the Adolescent SRH Stigma Scale comprised 20 items measuring three primary domains of environmental stigma (Internalized Stigma 6-items; Enacted Stigma 7-items; Stigmatizing Lay Attitudes 7-items). Specifically, these items reflected statements about environmental stigma, specifically regarding the disgrace and shame (internalized stigma), discrimination and marginalization (enacted stigma), and negative community norms (stigmatizing lay attitudes) that may occur with adolescent sex, pregnancy, childbearing, abortion, STI and family planning. Response options were on a 3-point Likert scale (disagree, neutral, agree). For our analyses, we created an additive index, whereby responses of “agree” were coded as (1) and summed for a total score, with scores ranging from 0–20 and higher scores indicating higher levels of perceived stigma.

#### Health and social wellbeing

We examined a series of items on physical and mental health and social wellbeing as potential predictors of SRH stigma. For health histories, women were asked whether they “suffer from any chronic medical condition or ongoing health problem, for example high blood pressure or asthma,” which we examined as a binary variable (any chronic health condition vs. none). We assessed current general and mental health status with a series of standardized items. We first used the 5-point Likert item for self-rated overall health (excellent, very good, good, fair, or poor) and present results as excellent/very good vs. <very good health. We assessed mental health symptoms with single items adapted from validated depression, anxiety and stress scales. Via the Patient Health Questionnaire (PHQ), women were asked how often, on a 5-pt scale (1 = never, 2 = almost never, 3 = sometimes, 4 = fairly often, or 5 = very often), in the past month they had been bothered by the following depression and anxiety symptoms, respectively: 1) feeling sad, depressed, or hopeless, and 2) feeling excessive worry, nervous, anxious, or on edge.[25] With the same response options, they were asked about whether they, “felt stressed, as if you were unable to control the important things in your life or difficulties were piling up so high that you could not overcome them,” a single item which was adapted from the Perceived Stress Scale (PSS).[26]

For social wellbeing, we administered the abbreviated Everyday Discrimination Scale.[27] Via five items, women were asked how often in their day-to-day lives (6-point likert scale: 1 = never, 2 = less than once a year, 3 = a few times a year, 4 = a few times a month, 5 = at least once a week, or 6 = almost every day) they had the following discrimination experiences: 1) “you are treated with less courtesy or respect than other people;” 2) “you receive poorer service than other people at restaurants or stores;” 3) “people act as if they think you are not smart;” 4) “people act as if they’re afraid of you;” and 5) “you are threatened or harassed.” Responses to the five discrimination items were summed for a total score, ranging from 0–25, with higher scores indicating greater discrimination symptoms. Finally, for other stressful social experiences, we adapted adverse life event (ALE) items used in prior studies of general and reproductive health [28,29]. These items measured 9 different ALEs, including financial, emotional, traumatic and partner-related experiences in one’s lifetime. Responses were binary (yes vs. no) and affirmative responses were summed for a total score, ranging from 0–9, with higher scores indicating more ALEs.

#### Sociodemographics and reproductive history

We examined the following sociodemographic variables as “predictors” of SRH stigma: age, ethnic group (Akan, Ga/Dangme, Ewe, vs. Other), religious affiliation (Muslim vs. Christian/Other), religious importance (extremely/very vs. not at all/somewhat/important), frequency of religious service attendance (≥weekly vs. <weekly), educational attainment (completed secondary school or more vs. <secondary school), relationships status (married/engaged, romantic/sexual relationship, vs. none), employment status (employed in last seven days vs. not employed) and health insurance status (insured vs. uninsured). We examined the following sexual and reproductive history characteristics: sexual intercourse with male partner (lifetime and in last 12 months, yes vs. no), number of sex partners (lifetime and last 12 months, 0, 1, vs. ≥2), sexual assault (lifetime and last 12 months, yes vs. no), physical assault by an intimate partner (lifetime, yes vs. no), receipt of family planning services (lifetime and last 12 months, yes vs. no), contraceptive use (lifetime and at last sex, yes vs. no), and histories of pregnancy, live birth, miscarriage/stillbirth, and abortion (lifetime, yes vs. no).

### Statistical analysis

Our analytic sample included 1,063 of the 1,080 participants (98.5%) who completed 60% or more of survey items, including all SRH stigma items. We used descriptive (means with standard deviations (SD), frequencies with proportions (%)) and bivariate statistics (Student’s T-test, ANOVA) to describe and compare SRH stigma scores across sociodemographic, health, and reproductive history groups. We used multivariable linear regression with a recruitment site cluster effect to simultaneously examine all available sociodemographic, health, and reproductive history factors potentially associated with stigma. Variables were considered for inclusion in regression models if their p-values in bivariate analyses were <0.25. We employed a step-wise approach, modeling sociodemographics first, then adding health and social wellbeing factors, followed by reproductive history factors. For factors that were highly collinear (e.g. sexual history variables, mental health variables), we retained those with the strongest effects in final models. We present regression results as adjusted beta coefficients (β) and 95% CIs. We used STATA 13.0 (College Station, TX) for all analyses.

## Results

Characteristics of the sample are presented in Table 1. The mean age of participants was 20 years. Equal proportions were from Kumasi and Accra. Half (52%) self-identified as Akan ethnicity. The predominant religious affiliation was Christian/other (88%), with 77% reporting religion as extremely/very important and 80% attending religious services weekly or more frequently. Less than half had completed secondary school (42%) and three-quarters (74%) were not employed. Nearly half were in a romantic or sexual relationship (47%), though few were married or engaged (15%). For health and social wellbeing, 37% rated their health as ≤ good; 8% had a history of chronic health condition. Current depression, anxiety and stress symptoms were reported, on average, one to two times per month (means 2.40, 2.19, and 2.09, respectively, on 1–5 scale). Mean scores on the everyday discrimination and stressful life events scales were 4.46 (range 0–25) and 3.07 (range 0–9), respectively. For sexual and reproductive histories, intercourse experience (lifetime 69%; last 12 months 63%) was common, with 43% reporting ≥ 2 lifetime partners. Half (52%) reported ever use of contraception; 41% had used contraception at last sex. Receipt of formal family planning services was less common (35%). Half had ever been pregnant (49%); a quarter had given birth (26%) and 10% reported an abortion history. Nearly one-third reported a history of sexual assault (30%).

**Table 1:** Characteristics of the sample.

(N = 1,063)	Mean or Proportion	SD or Frequency
*Sociodemographic Characteristics*		
Age (in years, range 15–24)	19.93	2.69
Ethnic group		
Akan	0.52	553
Ga/Dangme	0.14	144
Ewe	0.13	142
Other	0.21	222
City		
Kumasi	.50	527
Accra	.50	536
Religious Affiliation		
Muslim	0.12	129
Christian/Other	0.88	934
Religious importance		
Extremely/very important	0.77	821
Important/somewhat important/not at all important	0.23	240
Frequency of religious service attendance		
Attendance ≥ weekly	0.80	847
Attendance <weekly	0.20	216
Educational attainment		
Completed secondary school or more	0.42	445
< secondary school	0.58	618
Employment status		
Employed in last seven days	0.26	281
Unemployed	0.74	781
Relationship status		
Married or engaged	0.15	162
In a romantic or sexual relationship	0.47	497
Not in a relationship	0.38	402
Insurance status		
Insured	0.76	807
Uninsured	0.24	256
*Health & Social Wellbeing*		
Self-rated health		
Excellent/very good	0.63	665
Good/fair/poor	0.37	398
Health history		
Any chronic health conditions	0.08	83
No chronic health conditions	0.92	922
Depression symptoms (5-pt Likert scale, possible range 1–5)^a^	2.40	1.21
Anxiety symptoms (5-pt Likert scale, possible range 1–5)^b^	2.19	1.17
Stress symptoms (5-pt Likert scale, possible range 1–5)^c^	2.09	1.20
Everyday discrimination (mean score, possible range 0–25)^d^	4.46	4.82
Stressful life events (mean score, possible range 0–9)^e^	3.07	1.85
*Sexual & Reproductive History*		
Sexual intercourse with male partner		
Ever had sex	0.69	725
Never had sex	0.31	331
Sexual assault		
Ever been sexually assaulted	0.30	315
Never been sexually assaulted	0.70	739
Physical abuse by intimate partner		
Ever been physically hurt	0.16	167
Never been physically hurt	0.84	888
Receipt of family planning services		
Ever received services	0.35	369
Never received services	0.65	683
Contraceptive use		
Ever used contraception	0.52	533
Never used contraception	0.48	492
Pregnancy		
Ever pregnant	0.49	522
Never pregnant	0.51	541
Live birth		
Ever had live birth	0.26	275
Never had live birth	0.74	788
Miscarriage/stillbirth		
Ever had a miscarriage/stillbirth	0.05	51
Never had a miscarriage/stillbirth	0.95	1012
Abortion		
Ever had an abortion	0.10	111
Never had an abortion	0.90	952
Lifetime number of sex partners		
0 partners	0.31	331
1 partner	0.26	272
2+ partners	0.43	460
Had sex with male partner in last 12 months		
Yes	0.63	663
No	0.37	393
Contraceptive use at last sex		
Yes	0.41	254
No	0.59	372
Sexually assaulted in last 12 months		
Yes	0.09	95
No	0.91	959
Received family planning services in last 12 months		
Yes	0.22	235
No	0.78	817
Number of sex partners in last 12 months		
0 partners	0.39	341
1 partner	0.50	437
2+ partners	0.11	96

N = 1,063. Results presented as means with standard deviations or proportions with frequencies.

Responses to individual Likert items measuring depression,^a^ anxiety^b^ and stress^c^ symptoms (from the Patient Health Questionnaire and Perceived Stress Scale): 1 = Never/Not at all; 2 = Only 1–2 times per month/Almost never; 3 = 3–4 times per month/Sometimes/; 4 = At least once a week/Fairly often; 5 = Almost every day/ Very often.

^d^Mean discrimination score measured via abbreviated 5-Likert-item Everyday Discrimination Scale (possible range 0–25).

^e^Mean stressful life events score measured via 14-item additive index scale (range 0–14).

### Sexual and reproductive health stigma

Responses to the SRH Stigma Scale, sub-scales, and individual items are presented in Table 2. The mean score on the SRH Stigma scale was 13.11 (SD 3.78, range 1–20), with mean sub-scale scores highest for enacted stigma (4.55, SD 1.82, range 0–7) and similar for internalized (4.28, SD 1.42, range 0–6) and stigmatizing lay attitudes (4.28, SD 1.47, range 0–7). Agreement with the various stigma items was common, with 15 of the 20 items having >60% agreement. Stigma of abortion (64–92%), sex (56–86%), and childbearing/pregnancy (49–78%) had the highest rates of agreement while family planning stigma had the lowest agreement (31%-65%) (Table 2).

**Table 2:** Sexual and reproductive stigma scale, sub-scale, and item descriptives.

	Agree % (n)	Neutral/Disagree % (n)
**Full SRH Stigma Scale** (mean = 13.11 ± SD 3.78)		
**Enacted Stigma Sub-Scale** (mean = 4.28 ± SD 1.42)		
**1.** People behave differently toward a teen who they know has had sex	75.61 (803)	24.39 (259)
**2.** People behave differently toward a teen who they know has had an abortion	82.29 (869)	17.71 (187)
**3.** People behave differently toward a teen who they know has used modern family planning methods	61.25 (648)	38.75 (410)
**4.** Having sex as a teen often leads to getting beat or physically hurt by one's parents	56.21 (593)	43.79 (462)
**5.** Becoming pregnant and having a baby as a teen would cause people to behave differently around me	73.94 (783)	26.06 (276)
**6.** Becoming pregnant and having a baby as a teen would cause others to tease, insult, swear or gossip about me.	78.73 (829)	21.27 (224)
**Internalized Stigma Sub-Scale** (mean = 4.55 ± SD 1.82)		
**7.** Having sex as a teen is a form of disobedience	71.52 (751)	28.48 (299)
**8.** Young women who have abortions are bad girls	68.70 (722)	31.30 (329)
**9.** Young women who use modern family planning are promiscuous	45.37 (480)	54.63 (578)
**10.** Teens who use modern family planning are viewed as bad girls	65.37 (691)	34.63 (366)
**11.** Having sex as a teen brings disgrace and shame to a young woman and her family	64.97 (690)	35.03 (372)
**12.** Becoming pregnant and having a baby as a teen would bring disgrace to my family	70.96 (750)	29.04 (307)
**13.** Becoming pregnant and having a baby as a teen would make me feel ashamed and bad about myself.	68.56 (724)	31.44 (332)
**Stigmatizing Lay Attitudes Sub-Scale** (mean = 4.28 ± SD 1.47)		
**14.** Young women who have abortions will encourage others to have abortions	64.24 (679)	35.76 (378)
**15.** Modern family planning is not acceptable for unmarried women	31.39 (333)	68.61 (728)
**16.** Modern family planning methods have bad effects on a woman's health	46.37 (492)	53.63 (569)
**17.** Having an abortion is committing murder	91.56 (965)	8.44 (89)
**18.** The media, including the television, internet, or magazines, has a strong impact on teens' sexual behavior	86.33 (916)	13.67 (145)
**19.** When teens have sex for the first time, it is usually because they were pressured by their friends or partners to do so.	58.83 (623)	41.17 (436)
**20.** Children born to teen parents are worse off than those born to adults	49.01 (519)	50.99 (540)

N = 1,063. Results presented as frequencies (n) and proportions (%) for scale item descriptives and as means with standard deviations (SD) for overall scale and sub-scale scores.

### Factors associated with SRH stigma

In the unadjusted analysis (Table 3), factors associated with SRH stigma scores included: age, city, religious affiliation, educational attainment, relationship status, self-rated health, and histories of sexual intercourse, receipt of family planning services, modern contraceptive use, pregnancy, and number of sexual partners (p-values<0.05). Compared to their counterparts, SRH stigma scores were higher among girls who were younger, residing in Accra, Muslim religious affiliation, still in or had dropped out of secondary school, unemployed, in excellent/very good self-rated health, and who reported prior pregnancy(ies) and higher numbers of sexual partners (Table 3). On the other hand, SRH stigma scores were lower among girls who were in a relationship and those who had sexual intercourse experience, received family planning services, and used contraception (Table 3).

**Table 3:** Factors associated with sexual and reproductive health stigma in unadjusted analyses.

*Sociodemographic Characteristics*	Mean stigma score or unadjusted β coefficient	*p-value*
Age	-0.28	0.000
Ethnic group		
Akan	12.94	0.421
Ga/Dangme	13.20	
Ewe	13.43	
Other	13.31	
City		
Accra	13.54	0.000
Kumasi	12.69	
Religious affiliation		
Muslim	13.73	0.046
Christian/Other	13.03	
Religious importance		
Religion extremely/very important	13.20	0.160
Religion important/somewhat important/not at all important	12.81	
Religious services attendance		
Weekly or more frequently	13.17	0.318
<weekly	12.88	
Educational attainment		
Completed secondary school or more	12.12	0.000
Less than secondary school	13.83	
Employment status		
Employed in last seven days	12.72	0.044
Not employed	13.25	
Relationship status		
Married or engaged	12.65	0.000
In a romantic or sexual relationship	12.70	
Not in a relationship	13.80	
Health insurance status		
Insured	13.18	0.300
Uninsured	12.90	
*Health & Wellbeing*		
Self-rated health		
Excellent/very good	13.35	0.007
Good/fair/poor	12.71	
Health history		
Any chronic health conditions	13.40	0.492
No chronic health conditions	13.10	
Depression symptoms^a^	-0.12	0.199
Anxiety symptoms^b^	-0.02	0.834
Stress symptoms^c^	-0.18	0.067
Everyday discrimination^d^	0.01	0.710
Stressful life events^e^	0.11	0.069
*Sexual & Reproductive History*		
Ever had sex with male partner		
Yes	12.86	0.003
No	13.60	
Ever sexually assaulted		
Yes	12.88	0.235
No	13.18	
Ever physically hurt by intimate partner		
Yes	13.27	0.501
No	13.06	
Ever received family planning services		
Yes	12.71	0.015
No	13.31	
Ever used modern contraception		
Yes	12.75	0.002
No	13.50	
Ever pregnant		
Yes	13.35	0.043
No	12.88	
Ever had a live birth		
Yes	13.20	0.665
No	13.08	
Ever had a miscarriage/stillbirth		
Yes	12.53	0.260
No	13.14	
Ever had an abortion		
Yes	13.01	0.774
No	13.12	
Lifetime number of sex partners		
0 partners	13.60	0.000
1 partner	13.42	
2+ partners	12.58	
Had sex with male partner in last 12 months		
Yes	12.91	0.041
No	13.40	
Contraceptive use at last sex		
Yes	13.05	0.194
No	12.66	
Sexually assaulted in last 12 months		
Yes	13.34	0.497
No	13.07	
Received family planning services in last 12 months		
Yes	12.65	0.040
No	13.23	
Number of sex partners in last 12 months		
0 partners	13.61	0.074
1 partner	12.98	
2+ partners	13.08	

N = 1,063. Results presented as mean stigma scores or unadjusted beta (β) coefficients. P-values comparing stigma across sociodemographic, health and reproductive history groups via t-tests and ANOVA for binary/categorical predictors and bivariate regression for continuous predictors. P-values <0.05 considered significant. P-values <0.25 considered for inclusion in multivariable regression analyses.

Responses to individual Likert items measuring depression,^a^ anxiety^b^ and stress^c^ symptoms (from the Patient Health Questionnaire and Perceived Stress Scale): 1 = Never/Not at all; 2 = Only 1–2 times per month/Almost never; 3 = 3–4 times per month/Sometimes/; 4 = At least once a week/Fairly often; 5 = Almost every day/ Very often.

^d^Mean discrimination score measured via abbreviated 5-Likert-item Everyday Discrimination Scale (possible range 0–25).

^e^Mean stressful life events score measured via 14-item additive index scale (range 0–14).

In multivariable linear regression analyses (Table 4), factors positively associated with higher SRH stigma scores included history of pregnancy (β = 1.53, 95% CI = 0.51,2.56) and reporting excellent/very good (vs. ≤ good) self-rated health (β = 0.89, CI = 0.20,1.58). Older age (β = -0.17, CI = -0.24,-0.09), higher educational attainment (secondary school or greater vs. still in school/school drop-out, β = -1.22, CI = -1.82,-0.63), and sexual intercourse experience (β = -1.32, CI = -2.10,-0.55) were associated with lower SRH stigma scores.

**Table 4:** Multivariable linear regression models of factors predicting sexual and reproductive health stigma.

	Model 1			Model 2			Model 3			Model 4		
	Adj. β	(95% CI)		Adj. β	(95% CI)		Adj. β	(95% CI)		Adj. β	(95% CI)	
*Sociodemographic Characteristics*												
Age	-0.12	(-0.28, 0.05)		-0.12	(-0.28, 0.04)		-0.14	(-0.29, 0.00)		-0.17	(-0.24, -0.09)	^***^
City												
Kumasi	-0.68	(-1.83, 0.47)		-0.60	(-1.86, 0.67)		-0.98	(-2.10, 0.15)		-0.83	(-1.69, 0.02)	
Accra	ref			ref			ref			Ref		
Religious Affiliation												
Muslim	0.41	(-1.27, 2.09)		0.37	(-1.09, 1.83)		0.15	(-1.28, 1.58)				
Christian/Other	ref			ref			ref					
Religious importance												
Religion extremely/very important	0.54	(-0.17, 1.25)		0.36	(-0.28, 1.00)		0.48	(-0.12, 1.09)				
Religion important/somewhat/not important	ref			ref			ref					
Educational attainment												
Completed secondary school or more	-1.52	(-2.34, -0.70)	^**^	-1.55	(-2.42, -0.69)	^**^	-1.27	(-1.97, -0.57)	^**^	-1.22	(-1.82, -0.63)	^**^
Less than secondary school	ref			ref			ref			ref		
Employment status												
Employed in last seven days	-0.21	(-1.62, 1.19)		-0.23	(-1.71, 1.25)		-0.25	(-1.65, 1.15)				
Not employed	ref			ref			ref					
Relationship status												
Married or engaged	-0.50	(-2.00, 1.00)		-0.46	(-1.93, 1.01)		-0.78	(-2.24, 0.68)				
In a romantic or sexual relationship	-0.83	(-1.56, -0.09)	^*^	-0.80	(-1.46, -0.14)	^*^	-0.78	(-1.70, 0.14)				
Not in a relationship	ref			ref			ref					
*Health & Wellbeing*												
Self-rated health												
Excellent/very good				0.72	(0.06, 1.38)	^*^	0.79	(0.17, 1.41)	^*^	0.89	(0.20, 1.58)	^*^
<very good				ref			ref			ref		
^a^Depression symptoms				-0.11	(-0.53, 0.32)		-0.11	(-0.51, 0.30)				
^b^Stress symptoms				-0.05	(-0.37, 0.28)		-0.03	(-0.32, 0.26)				
^c^Stressful life events				0.08	(-0.18, 0.34)		0.05	(-0.23, 0.32)				
*Sexual & Reproductive History*												
Ever had sexual intercourse with male partner							-0.59	(-1.60, 0.41)		-1.32	(-2.10, -0.55)	^**^
Never had sex							ref			ref		
Ever sexually assaulted							-0.20	(-0.50, 0.10)				
Never sexually assaulted							ref					
Ever received family planning services							-0.39	(-0.97, 0.18)				
Never received services							ref					
Ever used modern contraception							-0.28	(-1.21, 0.66)				
Never used modern contraception							ref					
Ever pregnant							1.86	(0.81, 2.90)	^**^	1.53	(0.51, 2.56)	^**^
Never pregnant							ref			ref		
R^2^	0.09			0.10			0.13			0.11		
N	1057			1053			1005			1055		

*p<0.05.

**p<0.01.

***p<0.001 (two-tailed tests).

Notes: Results presented as adjusted beta (β) coefficients with 95% confidence intervals from multivariable linear regression models adjusting for cluster effect for recruitment site and fixed effect for city. M3 and M4 results are similar when ever sex with male partner is replaced by number of lifetime sex partners.

^a-b^Responses to individual Likert items measuring depression and stress symptoms (from the Patient Health Questionnaire and Perceived Stress Scale): 1 = Never/Not at all; 2 = Only 1–2 times per month/Almost never; 3 = 3–4 times per month/Sometimes/; 4 = At least once a week/Fairly often; 5 = Almost every day/ Very often.

^c^Mean stressful life events score measured via 14-item additive index scale (range 0–14).

## Discussion

Using a new validated Adolescent SRH Stigma Scale we developed and tested among adolescents in two urban, western Sub-Saharan African communities, we found high levels of environmental SRH stigma, especially reported by the youngest girls. Our nuanced measurement approach highlighted varying degrees of negative social and community norms, attitudes and beliefs occurring across different dimensions of SRH. While high levels of abortion stigma were not surprising, similar levels of abortion stigma reported for sexual intercourse and pregnancy–two distinct reproductive events that have been less studied in stigma research—were perhaps less expected. Building upon prior stigma work in the U.S. and Africa largely focused on HIV and abortion stigma, our findings add estimates of environmental SRH stigmas across a broader spectrum of reproductive life experiences. They also provide insight into SRH stigma specifically perceived by adolescents—a vulnerable but understudied group in African family planning and stigma research, despite the severe impact of stigma and its health and social consequences for this group [30–32].

We explored a diverse set of demographic, health, and social context factors associated with adolescent SRH stigma. Prior pregnancy was perhaps the clearest, strongest predictor among our young Ghanaian sample. This finding is consistent with those of studies (ours and others’) in which previously pregnant or parenting adolescents, both in U.S. and African cohorts, have reported discrimination, marginalization, and violence [15–17,33,34]. However, this relatively modest body of work has not explicitly conceptualized or formally measured SRH stigma. Also, congruent with our larger project in which we postulated that SRH stigma serves as a barrier to family planning, higher levels of stigma here were reported among girls who had never used contraceptive methods or services, though this trend was not significant in multivariable analyses. On the other hand, prior sexual intercourse experience was associated with *lower* levels of stigma. It is not clear from our data whether girls had disclosed prior sexual experiences, such that sex itself may not have been a stigmatizing event but rather the perceived social manifestations and consequences of it were. Alternatively, it may be that sex without the consequence of pregnancy could foster stigma resilience, or even a sense of pride or accomplishment. This requires further study. Of note, our survey items were designed to measure SRH stigma within women’s social environments and not (necessarily) experienced stigma; although in this analysis we explicitly assessed girls’ histories of a range of SRH events. Our ongoing work in Africa and the U.S. is further investigating stigma directly *attributable* to sex, pregnancy, contraceptive use, abortion and family planning service use.

Additionally, given the cross-sectional nature of our data, we were unable to establish temporality or examine time-varying levels of SRH stigma that may change over time as a result of new exposures. It is likely that stigma and SRH perceptions and experiences are bi-directionally associated–that one’s perceptions of SRH shaped by the SRH experiences of one’s own, one’s friends, family, or community members, which in turn may or may not be accompanied by stigma [22–24]. Conversely, one’s SRH experiences may be shaped by negative attitudes and beliefs about SRH, which further perpetuate stigma. Additional longitudinal studies are needed to disentangle the complex, dynamic interrelationships between adolescents SRH perceptions, experiences and stigma.

Regarding social and health predictors, lack of education, unemployment, and religious affiliation (Muslim) were associated with adolescent SRH stigma, findings which align with results of other studies documenting high levels of reproductive and other types of stigmas among vulnerable or conservative groups [1–11,15–17,33,34]. Social discrimination, depression, anxiety, and physical health conditions were not associated with stigma, although psychological stress and adverse life events were marginally associated. Perhaps other stigmatized identities, in this case mental or physical illness or other factors contributing to discrimination, foster stigma resilience. It is also possible that while discrimination and mental health morbidity have historically been conceptualized as common correlates of stigma [1–11], perhaps they do not contribute to *SRH* stigma specifically, or even to stigma in this geographic/cultural context. In our longitudinal analysis of a cohort of adolescent girls in the United States [34], we found that history of pregnancy was the strongest predictor of everyday discrimination and of stress but not depression; stigma was not measured. For the current study, it is also possible that other confounding factors not comprehensively measured, such as violence exposure, relationship dynamics, or health, account for associations of interest (or lack thereof). Moreover, to our knowledge, items from the Everyday Discrimination Scale and the Patient Health Questionnaire have not been widely validated among African or adolescent samples, and we adapted abbreviated versions of these scales which offer limited measurement of relatively complex health and social constructs. Overall, the interrelationships between social wellbeing, physical and mental health, and SRH stigma require further investigation using more holistic bio-psycho-social frameworks and methodological approaches.

In addition to the above-described limitations, our results were likely impacted by social desirability bias given the sensitive nature of our study and our young sample. Additionally, we focused on two major cities in Ghana and on participants recruited from schools and health facilities, such that our findings are not generalizable to all girls in Sub-Saharan Africa or other sociocultural or geographic contexts in which SRH stigma may be perceived or experienced differently. Although, the SRH stigma found here is perhaps notable given the relatively developed reproductive healthcare infrastructure in Ghana compared to other countries. We did not explore the perceptions or experiences of SRH stigma and its predictors among adolescent boys or of transgendered youth, which are highly relevant sub-populations for which SRH stigma has been understudied and deserve future research inquiry. Finally, our stigma scale did not capture the full spectrum of SRH stigmas, for example HIV, STIs or sexual minority stigmas, or an exhaustive list of potential predictors of stigma.

Nonetheless, our study suggests that these adolescent girls endorse stigmatizing attitudes around issues of SRH and/or have observed or felt SRH stigma in their environments. Some life experiences or lack thereof appear to render some girls more vulnerable to stigmatizing beliefs (e.g. no prior health challenges, having had a pregnancy, never having had sex), and others more resilient to stigma (e.g. being sexually active but successfully avoiding pregnancy). While our findings provide insight into a diverse set of factors associated with SRH stigma in Ghana, they also highlight clear remaining gaps in adolescent health research and important directions for future study. Research is needed to inform more holistic, evidence-based practices, programs and policies that can foster stigma resilience at the individual level and counter adolescent SRH stigma at the environmental level in order to improve SRH outcomes for young women and communities across the globe.
